# Organic heterojunctions for direct solar fuel generation

**DOI:** 10.1038/s42004-020-0288-z

**Published:** 2020-03-27

**Authors:** Reiner Sebastian Sprick, Marc A. Little, Andrew I. Cooper

**Affiliations:** Department of Chemistry and Materials Innovation Factory, University of Liverpool, Liverpool, L7 3NY United Kingdom

**Keywords:** Photochemistry, Polymer chemistry, Polymers, Photocatalysis

## Abstract

Organic polymers have demonstrated promise as photocatalysts, but their photocatalytic efficiencies remain relatively low. Now, borrowing principles from organic photovoltaics, heterojunctions of polymer photocatalysts and small molecule acceptors have been shown to have excellent solar hydrogen production efficiencies.

There has been a surge of interest over the last decade in organic photocatalysts for solar fuel production^1^. Hydrogen production from water has been studied extensively: in particular, direct photochemical water splitting has been touted for its technological simplicity—no metal contacts or wiring is required—which could make it potentially scalable, if solar efficiencies can be improved. Traditionally, inorganic semiconductors have dominated the field of photocatalysis, but the discovery of carbon nitride photocatalysts in 2009 by Xinchen Wang and co-workers provoked intense interest in soft organic materials as photocatalysts^2^. Many carbon nitride based materials followed, as well as other organic materials with more well-defined structures, such as conjugated polymers, conjugated microporous polymers, and covalent organic frameworks. All of these materials have been reported to act as photocatalysts for hydrogen production from water in the presence of hole-scavengers^1^.

The key challenge for organic polymer photocatalysts is the relatively low photocatalytic efficiency compared to inorganic semiconductors. In part, this is due to the high exciton binding energies in organic materials—typically on the order of tenths of electronvolts—that result from the Coulombic attraction between the negative and positive charges that are generated after light absorption. Organic semiconductors also have comparatively poor charge-transport, which results in short exciton diffusion lengths and poor exciton separation. Hence, most charges generated in these organic solids will not reach the surface and reduce protons, particularly in the case of micron-sized particles, which results in low overall efficiencies because charges are lost due to recombination.

Another problem is that most of the organic photocatalysts investigated so far do not absorb light where the output of the Sun that reaches the Earth’s surface is strongest; indeed, most materials reported only absorb visible light up to around 500 nm. While far infrared light does not have the required energy to facilitate water splitting, many photons in the red region are not used to generate hydrogen by the organic catalysts reported thus far.

Recently, McCulloch and co-workers overcame these limitations by using conjugated polymer/non-fullerene acceptor heterojunction nanoparticles^3^. The energy off-set of the conjugated polymer (**PTB7-Th**) relative to the acceptor molecule (**EH-IDTBR**) results in exciton separation at the interface. This approach has been used extensively in organic photovoltaic devices^4^ (Fig. 1a) and it was found to be highly effective here for photocatalytic hydrogen production. Control of the interface and mixing was found to be crucial, and core-shell nanoparticles were found to be inefficient. A transition to heterojunctions was made by controlling the polymer–acceptor interface (Fig. 1b). Much higher photocatalytic activities were observed for these heterojunction photocatalysts compared to the core-shell photocatalysts after loading with a metal co-catalyst in the presence of a sacrificial hole scavenger (Fig. 2). While the so-called external quantum efficiencies (EQE) were relatively low (2% at 400 nm to 6.2% at 700 nm) compared to state-of-the-art organic photocatalysts (e.g., 22.8% at 420 nm for covalent triazine-based frameworks^5^), the light absorption was extended to the range of 500–750 nm for the **PTB7-Th**/**EH-IDTBR** heterojunctions, thus covering a much larger proportion of the visible light spectrum, which greatly enhances the performance of these heterojunction composites. This is an important step forward in the control of function for organic photocatalysts.

**Fig. 1:**
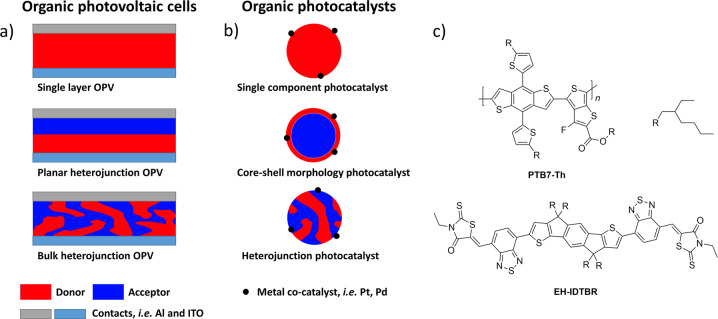
Different architectures that have been used in organic photovoltatic cells^4^. **a** Device architectures and **b** photocatalyst structures. **c** The photocatalyst system used by McCulloch and co-workers^3^.

**Fig. 2:**
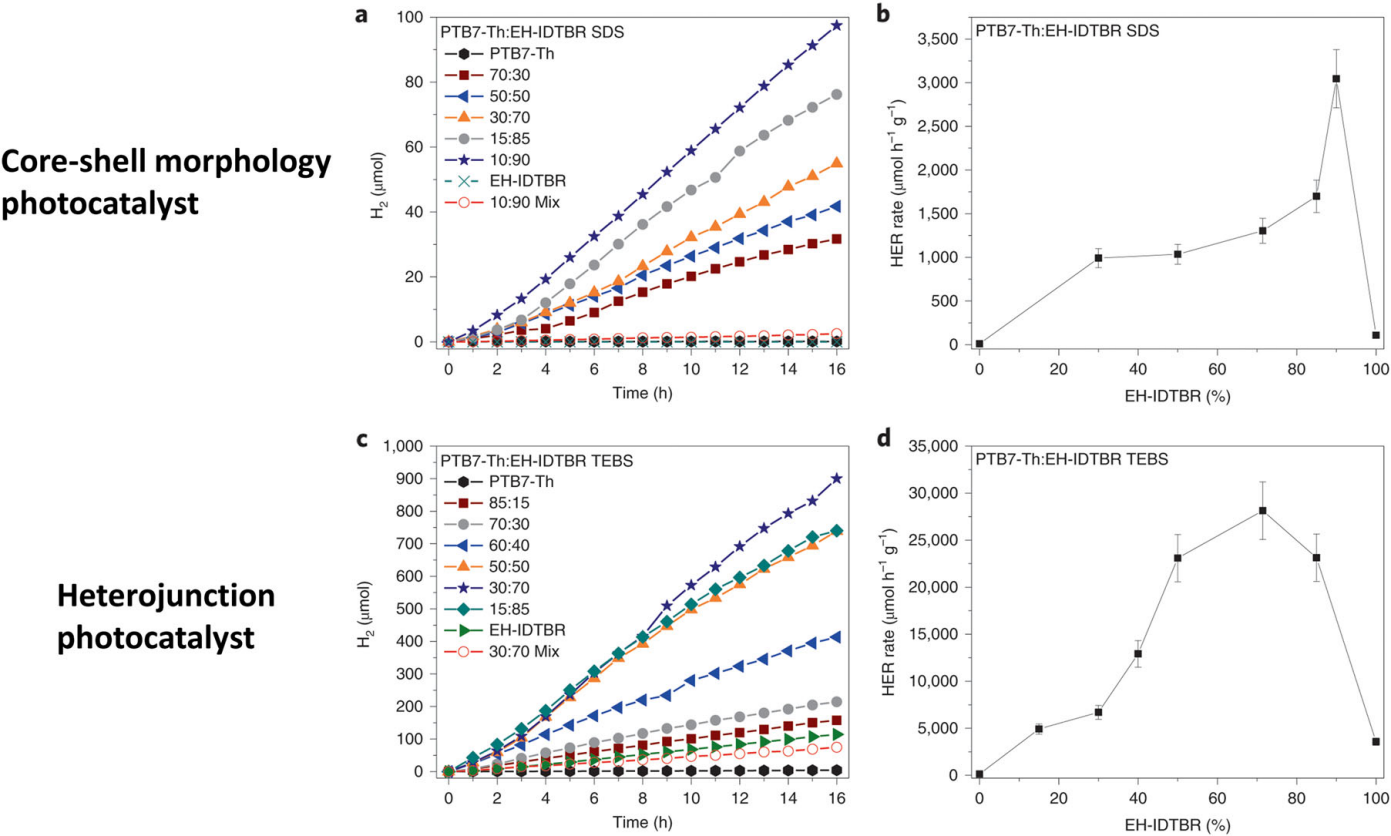
Hydrogen production using different types of photocatalyst morphologies. Photocatalytic activity of core-shell photocatalysts (**a**, **b**) and heterojunction photocatalysts (**c**, **d**) used by McCulloch and co-workers^3^. Reproduced with permission^3^. Copyright 2020, Nature Research.

Independently, we explored a very similar approach in creating heterojunctions of conjugated polymers/non-fullerene acceptors using a semi-automated robotic screening approach that allowed us to explore a large number of donor–acceptor combinations and different ratios, including ternary systems^6^. As in the work of McCulloch and co-workers, we also found that this approach leads to large improvements in activity for sacrificial hydrogen production from water. Similarly, we also used nanoparticles because it increases the area of the composite that is in contact with water and hole scavenger.

The organic heterojunction approach can, in principle, be extended to a whole range of other organic materials including covalent organic frameworks (COFs) and molecular organic crystals. COFs and molecular crystals are modular materials, meaning that they can be processed into co-crystals or solid solutions to tune their structures and functions. However, it remains challenging to alter structural features in these organic solids in a purposeful way to achieve superior function: for example, to favorably tune their light adsorption profile, charge-transport behavior, electronic conductivity, porosity, and wettability.

Recently, we have been studying the relationship between the structures of COFs and organic molecular crystals and their photocatalytic performance. Previously, we found that a porous fused sulfone-based 2D COF could be dye sensitised to improve its photocatalytic performance (**FS-COF**, Fig. 3a)^7^. By introducing a near-infrared absorbing dye into the pores of **FS-COF**, we increased the EQE of the COF from 0.6 to 2.2% at 600 nm. Likewise, **FS-COF** was completely inactive at 700 nm, while the COF composite had an EQE of 0.7%. The dye-sensitised COF showed a 61% enhancement in hydrogen production under visible light irradiation. The high photocatalytic rate for this material was ascribed to aligned stacking of 2D layers in **FS-COF**, but it is often difficult to control interlayer packing in COFs—also, single crystals of COFs are very rare^8^. By comparison, it is often straightforward to produce molecular crystals with high crystallinity, which offers exciting opportunities to deconvolute structure-property relationships at the atomistic level. For example, in a recent study, we found a molecular crystal (**TBAP**-α, Fig. 3b) that is the first example of a hydrogen-bonded organic framework (HOF) that shows appreciable photocatalytic hydrogen evolution from water under sacrificial conditions^9^. **TBAP**-α is highly porous, but we attribute the high proton reduction rate for this material more to the aligned stacking of conjugated pyrene cores than to its porosity. We screened for the crucial π-stacking motif in this study using in silico searches (Fig. 3c). Interestingly, the EQE for **TBAP**-α is 4.1% at 420 nm, which is higher than some conjugated polymer catalysts, even though this material lacks extended conjugated bonding.

**Fig. 3:**
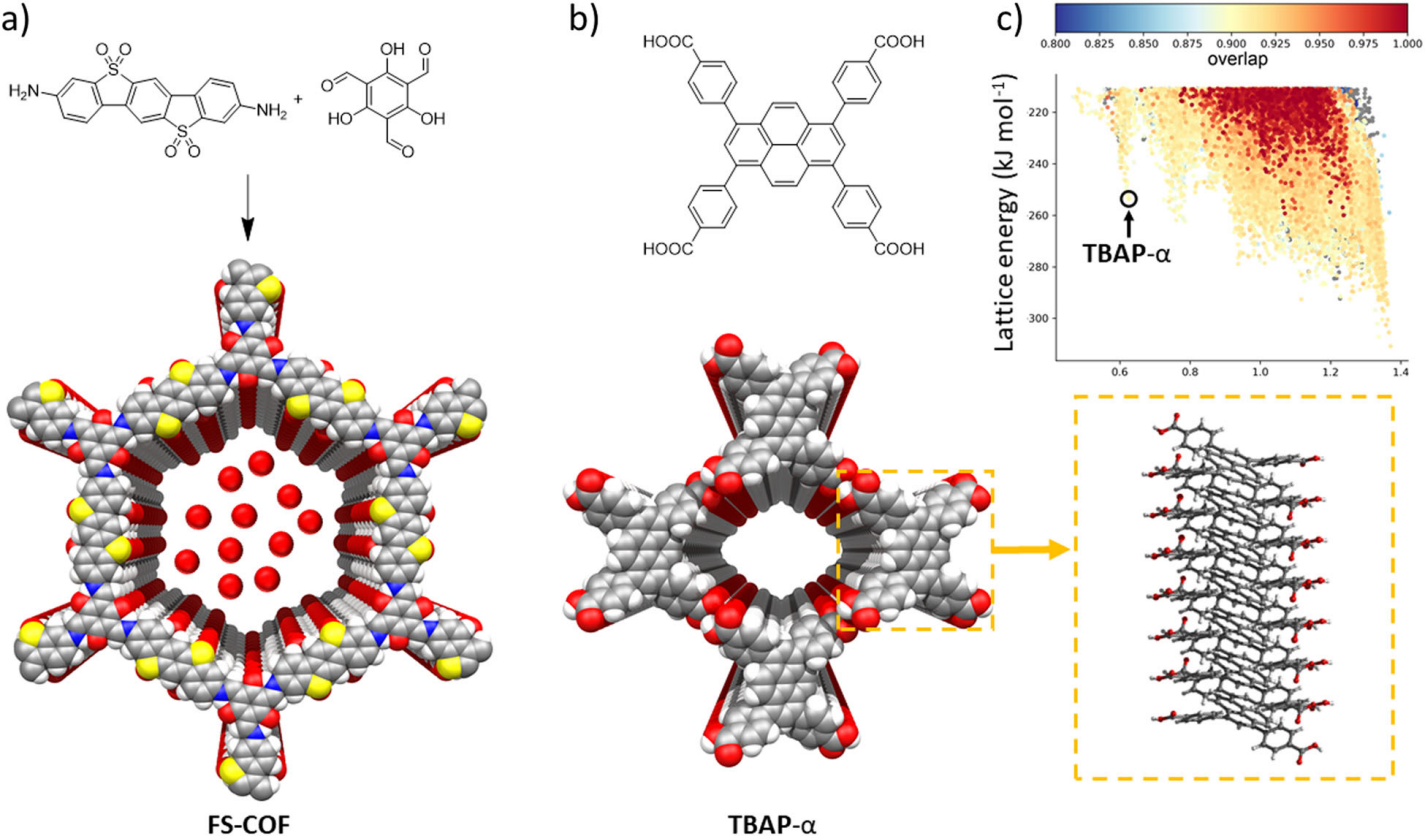
Dye-sensitised COF and molecular crystal photocatalysts. **a** Synthesis of **FS-COF**, which adsorbs a near-infrared absorbing dye to create a dye-sensitised COF that has an enhanced hydrogen production rate under visible light irradiation^7^. **b**, **c** Chemical structure of **TBAP**, which was predicted to form a porous hydrogen-bonded structure, **TBAP**-α, that features aligned stacks of pyrene along its pore walls and has a high hydrogen reduction rate under visible light irradiation. The colors in the plot denote the degree of pyrene overlap, or stacking, in the predicted crystal structures, showing that most low-energy predicted structures, such as **TBAP**-α, are dominated by pyrene stacks^9^.

In principle, crystal engineering with COFs and molecular crystals can be used to control the interface of donor/acceptor components with atomistic precision. It should, therefore, be possible to create heterojunctions of crystalline materials by combining two components, as in the polymer approach reported by McCulloch and co-workers, perhaps to create controlled, near-ideal interdigitated structures that give an even higher photocatalytic performance. The future looks bright, both for polymer heterojunctions and crystalline organic photocatalysts.

## Outlook

Organic heterojunctions have shown new potential for direct photocatalytic hydrogen production by borrowing principles from organic photovoltaics. These ideas might also be applicable to carbon dioxide reduction and nitrogen fixation. The ability to process organic materials at low temperatures into films offers the potential for future applications on a large scale, and also for the development of Z-schemes to facilitate overall water splitting without using scavengers.
